# Association of MASP-2 Levels and *MASP2* Gene Polymorphisms with Rheumatoid Arthritis in Patients and Their Relatives

**DOI:** 10.1371/journal.pone.0090979

**Published:** 2014-03-14

**Authors:** Isabela Goeldner, Thelma Skare, Angelica B. W. Boldt, Flavia R. Nass, Iara J. Messias-Reason, Shirley R. Utiyama

**Affiliations:** 1 Department of Medical Pathology, Federal University of Paraná, Curitiba, Brazil; 2 Rheumatology Unit, Evangelical University Hospital, Curitiba, Brazil; 3 Department of Clinical Analysis, Federal University of Paraná, Curitiba, Brazil; Northwestern University Feinberg School of Medicine, United States of America

## Abstract

**Background:**

Mannan-binding lectin-associated serine protease 2 (MASP-2) is a key protein of the lectin pathway of complement. MASP-2 levels have been associated with different polymorphisms within *MASP2* gene as well as with the risk for inflammatory disorders and infections. Despite its clinical importance, MASP-2 remains poorly investigated in rheumatoid arthritis (RA).

**Methods:**

In this case-control study, we measured MASP-2 serum levels in 156 RA patients, 44 patient relatives, and 100 controls from Southern Brazil, associating the results with nine *MASP2* polymorphisms in all patients, 111 relatives, and 230 controls genotyped with multiplex SSP-PCR.

**Results:**

MASP-2 levels were lower in patients than in controls and relatives (medians 181 vs. 340 or 285 ng/ml, respectively, P<0.0001). Conversely, high MASP-2 levels were associated with lower susceptibility to RA and to articular symptoms independently of age, gender, ethnicity, smoking habit, anti-CCP and rheumatoid factor positivity (OR = 0.05 [95%CI = 0.019–0.13], P<0.0001 between patients and controls; OR = 0.12, [95%CI = 0.03–0.45], P = 0.002 between patients and relatives; OR = 0.06, [95%CI = 0.004–0.73], P = 0.03 between relatives with and without articular symptoms). *MASP2* haplotypes *2A1 and *2B1-i were associated with increased susceptibility to RA (OR = 3.32 [95%CI = 1.48–7.45], P = 0.004). Deficiency-causing *p.120G* and *p.439H* substitutions were associated with five times increased susceptibility to articular symptoms in relatives (OR = 5.13 [95%CI = 1.36–20.84], P = 0.02). There was no association of MASP-2 levels or *MASP2* polymorphisms with autoantibodies, Sjögren's syndrome, nodules and functional class.

**Conclusions:**

In this study, we found the first evidence that MASP-2 deficiency might play an important role in the development of RA and articular symptoms among relatives of RA patients.

## Introduction

Rheumatoid arthritis (RA) is an autoimmune disorder with complex etiology which affects approximately 0.5–1.1% of the world population and has an annual incidence of 3 per 10 000 adults [1]. RA represents a substantial economic burden due to frequent hospitalizations, disability and productivity loss faced by the patients, culminating with 2–3 times increased mortality rates [2]. Despite the increasing number of studies aiming at the development of new RA therapy strategies, up to 50% RA patients do not achieve disease remission [3]. The failure to recognize the disease in early stages, as well as treatment side effects, are still the main obstacles to be overcome.

The disease is characterized by intense destructive inflammation promoted by immune deregulation. Similarly to other autoimmune diseases, multiple factors are known to contribute to its development. Recent genome-wide association studies have confirmed that genetic factors play an important role in the etiology of RA, but many of these factors need to be validated in the Brazilian population [4]–[6]. The important genetic contribution in the development of RA turns relatives of RA patients to be more susceptible to the disease [7]–[9]. Furthermore, familial RA aggregation is associated to autoantibody positivity and poorer disease prognosis [10], [11].

The complement system is an important arm of the innate immunity and plays a crucial role in the first line defense of the host. Complement activation has been shown to be an important mediator of inflammation in RA, being associated with severity of joint destruction [12]. The lectin pathway leads to complement activation through the pattern recognition molecules mannan-binding lectin (MBL) and ficolins, which in association with mannan-binding lectin-associated serine protease 1 and 2 (MASP-1 and -2), recognize a wide variety of microorganisms and cell debris, leading to complement activation and their clearance. Thus, MASPs play a relevant role in complement activation by the lectin pathway [13], [14]. So far investigations have been made aiming to clarify the role of MBL in the pathogenesis of RA [15]. Despite the importance of MASP-2 in the activation of complement, studies regarding its role in this context are still scarce [16]–[19].

MASP-2 is a protein analogous to the C1s and C1r serine proteases of the classical complement pathway [20]. It is composed of well-defined domains, including the CUB1 (C1r/C1s, Uegf and bone morphogenetic protein-1 domains), EGF (epidermal growth factor), CUB2, CCP1 (complement control protein 1), CCP2 and SP (serine protease) domains [21]. The *MASP2* gene is located on chromosome 1p36.3–36.2, in a region that has been found associated with RA and other inflammatory autoimmune conditions in recent studies [22]–[24]. Its single nucleotide polymorphisms (SNPs) are associated with different MASP-2 levels which are stable in adult healthy individuals but not in the pediatric population [25]–[27]. The *p.D120G* SNP is located in exon 3 of *MASP2* gene and affects the CUB1 domain and the binding of MASP-2 to ficolins or MBL. In the homozygous state, it determines MASP-2 deficiency in approximately 0.3% of European individuals [25], [28]. In addition, amino acid substitutions caused by *p.P126L* (first CUB domain), *p.V377A* (second CCP domain) and *p.R439H* (SP domain) are also known to reduce MASP-2 concentration and, in the case of *p.R439H*, impair complement activation [25]. We recently described ten *MASP2* haplotypes combined with five other SNPs distributed from the promoter to the last exon (5′untranslated region) [26].

Only few disease association studies on *MASP2* polymorphisms and MASP-2 levels have been reported so far, none in RA [29]–[32]. In the present study, we aimed to investigate whether MASP-2 serum levels and *MASP2* polymorphisms could influence the susceptibility to RA and its clinical presentation in a population from Southern Brazil. We found an association between low MASP-2 levels and deficiency-causing substitutions and RA, as well as an association between high MASP-2 levels and lower susceptibility to RA in relatives from patients.

## Materials and Methods

### Ethics statement

The Ethics Research Committee from Evangelical Beneficent Society, Curitiba, Brazil, approved this study and the experiments were performed in accordance with the Declaration of Helsinki. All subjects provided written informed consent, which was approved by the Ethics Committee.

### Subjects and samples

One-hundred and fifty-six adult RA patients from the urban population of Paraná's capital (Curitiba) were diagnosed according to the American College of Rheumatology criteria in the Rheumatological Unit of the Evangelical Hospital of Curitiba (Brazil) [33], and consecutively included in the study from August 2007 to April 2009 (84.6% female, 15.4% male, mean age 51.5 years, range 24 to 77 years, with 1–60 years of documented disease).

Clinical and demographic data were obtained from medical records and interviews using a standard questionnaire (Table 1). To access the extent of physical disability in RA patients, the Steinbrocker functional classification was applied. This index of disease activity rates on a four-level scale, ranging from class I, complete functional capacity, to class IV, largely or wholly incapacitated. Functional classes III and IV were grouped due to its low sample number. Detailed description of anti-CCP and rheumatoid factor determinations has been given elsewhere [9]. Two hundred thirty healthy unrelated individuals matched for age and gender with RA patients were used as a control group (82.6% female, 17.4% male, mean age 45.8 years, range 24 to 89 years). To reduce the potential for selection bias, we selected controls from different sources: 202 blood donors from three different blood banks in Curitiba (58 from the Hospital de Clínicas – UFPR, 144 from the Biobanco of the Hospital Evangélico) and 28 volunteers without RA from the Curitiba's surrounding (Witmarsum).

**Table 1:** Clinical and demographic data of RA patients.

Characteristic	Patients % (n = 156)
Anti-CCP positivity	72.3 (119)
Rheumatoid factor positivity	69.2 (108)
Females	84.6 (132)
Age at disease onset	
<40 years	39.1 (61)
≥40 years	60.9 (95)
Disease duration	
6 to 24 months	17.3 (27)
>2 to 10 years	51.9 (81)
>10 years	30.8 (48)
Steinbrocker functional class	
Class I	48.1 (75)
Class II	40.4 (63)
Class III	9.6 (15)
Class IV	1.9 (3)
Extra-articular manifestations	
Rheumatoid nodules (139)^a^	7.9 (11)
Sjögren syndrome (114)^a^	32.4 (37)

a : Total number of samples with available data for the extra-articular manifestation.

Patients, relatives and controls were defined as being from major European, African or Amerindian ancestry based on physical characteristics and informed ethnic background. Based on HLA allelic frequencies of South Brazilian population samples identified in the same way, Euro-Brazilians have an average sub-Saharan African component of 9% and an average Amerindian component of 5%, whereas Afro-Brazilians have at least 40% of African and 6% of Amerindian ancestry [34], [35]. Patients and control subjects had the same socioeconomic status and were from the same geographical area.

During the routine medical appointment of RA patients, their relatives were invited to participate on the study. From about 150 relatives invited, 111 (74%) relatives accepted to participate and had their blood samples collected (57.6%, 64/111 female, 42.4%, 47/11 male, mean age 38.5 years, range 7 to 73 years). Using a questionnaire and clinical examination, relatives were investigated for articular symptoms suggestive of RA (swollen or tender joints).

Venous blood was collected using anticoagulant and genomic DNA was extracted from mononuclear peripheral cells through Genomic Blood DNA Purification kit, GE Healthcare (São Paulo, Brazil). In addition, venous blood was collected without anticoagulant, centrifuged at 800 g^−1^ for 15 minutes, aliquoted and stored at −80°C until the assays for MASP-2 levels could be performed.

### MASP-2 levels measurement

We measured MASP-2 concentrations in sera of 156 RA patients, 44 patient relatives and 100 healthy individuals by ELISA according to the manufacturer's protocol (HK326 kit, Hycult Biotechnology, Uden, The Netherlands). For economic reasons, MASP-2 concentrations were determinate in randomly chosen subgroups of relatives and controls. The minimum measurable MASP-2 concentration of the ELISA kit used was 1.6 ng/ml. The color intensity was evaluated at 450 nm in an ELISA reader. Samples with a mean absorbance above the absorbance for the highest standard concentration were repeated using a higher dilution. MASP-2 levels lower than 200 ng/ml were considered low MASP-2 levels (cut-off adopted by others [26], [36]).

### *MASP2* haplotyping

We genotyped the following single nucleotide polymorphisms (SNPs) NG007289.1: *g.4847A>C* (SNP database: rs7548659), *p.R99Q* (rs61735600), *p.D120G* (rs72550870) and *p.P126L* (rs56392418) in exon 3, *g.21081C>T* (rs17409276) in intron 9, *p.D371Y* (rs12711521) and *p.V377A* (rs2273346) in exon 10, *p.R439H* (rs12085877) and *g.24762C>T* (rs1782455) in exon 12. These polymorphisms were previously associated with variation of MASP-2 levels [37]. The phase between the SNPs was determinate using sequence-specific primer polymerase chain reactions (PCR-SSP). A simple PCR-SSP was designed to identify the *p.D120G* mutation and the remaining SNPs were identified using two multiplex PCRs (I and II), as described [26]. Interpretation was based on the presence or absence of bands corresponding to the amplified fragments in agarose gel electrophoresis.

### Statistical analyses

Tests of independence between RA patients and the comparison groups were performed with binary logistic regression, using the Intercooled STATA 9.2 program (STATACorp). Using the same program, the number of included variables was gradually minimized, first eliminating those with a Wald's test p value higher than 0.20 and finally those with p values higher than 0.05 (whose contribution to final adjustment is almost insignificant), to end up with a reduced model of binary logistic regression. The comparison of MASP-2 levels between the groups was done through nonparametric Kruskal-Wallis or Mann-Whitney tests. Correlation analysis was done using Spearman correlation coefficient for nonparametric data. This statistical analysis was undertaken using the GraphPad Prism 3.0 software package (GraphPad Software Inc.). Two-tailed P-values less than 5% were considered significant. Direct counting was used to estimate genotype, allele and haplotype frequencies. SNPs distributed in the promoter and exon 3, intron 9, exon 10 and exon 12 could be phased with the SSP primers. The phase between distantly situated SNPs could be deduced due to strong linkage disequilibrium between the variants. Deviations from Hardy–Weinberg equilibrium and from the hypothesis of homogeneity between haplotype distributions (exact test of population differentiation of Raymond and Rousset) were tested using the ARLEQUIN software package version 3.1 (http://anthro.unige.ch/arlequin/). Linkage disequilibrium analysis (D′ and r2) between the *MASP2* SNPs was performed with the Haploview v.3.2 program (Broad Institute of MIT and Harvard).

## Results

### MASP-2 levels

**Figure 1:**
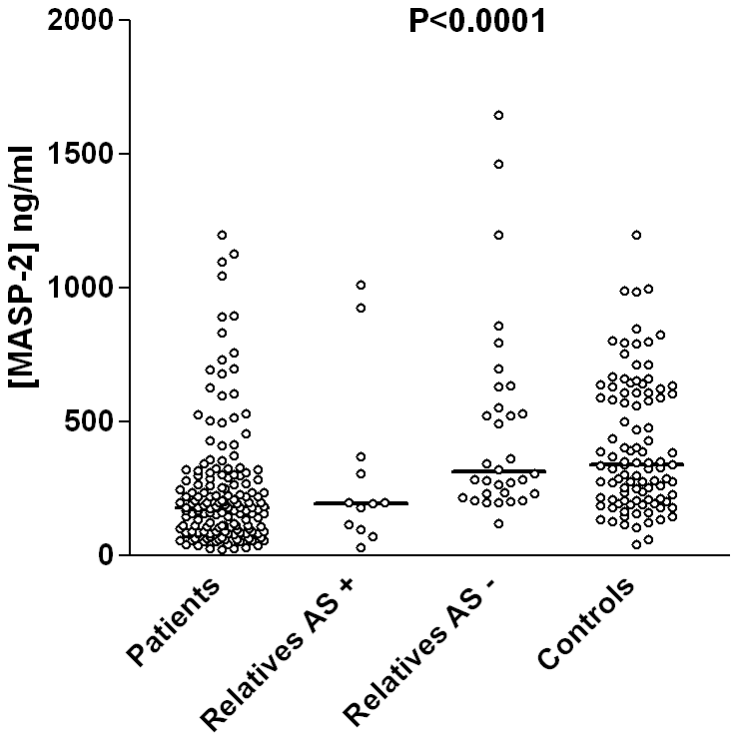
Differences between MASP-2 levels of patients, relatives with and without articular symptoms, and controls. Data is shown in medians and interquartile ranges, differences were calculated with Mann-Whitney tests (P<0.0001 between patients and controls, and patients and asymptomatic relatives; P = 0.0339 between symptomatic relatives and controls and P = 0.0143 between symptomatic and asymptomatic relatives). AS: articular symptom.

MASP-2 levels were significantly lower in RA patients than in controls and relatives (median 181 [21–1200 ng/ml, IQR = 199 ng/ml] vs. 340 ng/ml [42–1200 ng/ml, IQR = 398 ng/ml] and median 181 vs. 285 ng/ml [30–1646 ng/ml, IQR = 329 ng/ml], respectively, Mann-Whitney P<0.0001, Figure 1), but did not differ between patient groups with different clinical characteristics. On the other hand, high MASP-2 levels were associated with lower susceptibility to RA independently of age, gender and ethnicity (P<0.0001).

Whereas anti-CCP positivity increases almost seven times the susceptibility to RA between relatives, high MASP-2 levels decreases this susceptibility independently of age, gender, ethnicity, smoking habit and anti-CCP and rheumatoid factor positivity (Table 2).

**Table 2:** Reduced binary logistic regression models for rheumatoid arthritis.

Patients vs. Controls (P<0.00001)	n = 256	OR	95%CI	P
MASP-2		0.05	0.019–0.13	<0.0001
*2A1 and/or *2B1-i a		3.32	1.48–7.45	0.004

Patients vs. relatives (P<0.00001)	n = 200			
MASP-2		0.12	0.03–0.45	0.002
Anti-CCP positivity		6.45	2.38–17.45	<0.0001
Rheumatoid factor positivity		5.19	1.55–17.40	0.008
Age		1.04	1.004–1.074	0.026

Relatives with vs. without articular symptoms	&			
MASP-2 (P = 0.011)	n = 44	0.06	0.004–0.73	0.03
*1C2-l and/or *2B2B-l b (P = 0.024)	n = 111	5.13	1.26–20.84	0.02

The original model included age, gender and ethnicity for all observations; smoking habit, anti-CCP and RF positivity for patients and relatives. Significance of the model is given in parentheses. MASP-2 levels were normalized as logarithms on base 10 (Log_10_). anti-CCP: anti-cyclic citrullinated peptide antibodies; n: number of included observations; &: not independent; OR: odds ratio;

a : with intermediary levels,

b : with low MASP-2 levels.

In addition, relatives with articular symptoms had lower MASP-2 levels than controls, being similar to patients (median 196 ng/ml vs. 340 ng/ml, Mann-Whitney P = 0.0339, Figure 1), whereas relatives without those complaints presented similar values to the controls, i.e., with higher MASP-2 levels than patients (median 315.5 vs. 181 ng/ml, Mann-Whitney P<0.0001, Figure 1). There was also a difference in MASP-2 levels between relatives with and without articular symptoms (median 196 ng/ml vs. 315.5 ng/ml, Mann-Whitney P = 0.0143, Figure 1). High MASP-2 levels decreases the susceptibility to articular symptoms in otherwise healthy relatives, independently of age, gender, ethnic group, smoking habit and anti-CCP and rheumatoid factor positivity (Table 2). In contrast, anti-CCP and rheumatoid factor levels had no effect in the development of symptoms (P = 0.17 and 0.28, respectively, binary logistic regression) and did not correlate with MASP-2 levels (P = 0.9618, r = −0.0039 for anti-CCP and P = 0.3909, r = −0.0796 for rheumatoid factor, Spearman correlation coefficient test).

### MASP2 haplotypes

Genotype frequencies were in Hardy-Weinberg equilibrium. We found most of the pairs of evaluated SNPs to be in absolute linkage disequilibrium (data not shown) and identified eleven *MASP2* haplotypes in patients, relatives and healthy controls. One of them (*2B2A-i.1B1-h) is a recombinant haplotype identified for the first time in this study (Table 3).

**Table 3:** *MASP2* haplotypes nomenclature and frequencies (% ± SD).

Haplotype^a^	Human Genome Variation Society^b^	Controls n = 460	Relatives n = 222	Patients n = 312
*1A	[g.4847C; p.99R; p.120D; p.126P; g.21081C; p.371D; p.377V; p.439R; g.24762C]	5.2±1.0	6.3±1.6	4.8±1.2
*1B1-h	[g.4847C; p.99R; p.120D; p.126P; g.21081T; p.371D; p.377V; p.439R; g.24762C]	13.9±1.6	14.4±2.4	16.0±2.1
*1B2-h	[g.4847C; p.99Q; p.120D; p.126P; g.21081T; p.371D; p.377V; p.439R; g.24762C]	1.1±0.5	0	0.6±0.4
*1C1-l	[g.4847C; p.99R; p.120D; p.126L; g.21081C; p.371D; p.377V; p.439R; g.24762C]	1.3±0.5	0	0
*1C2-l	[g.4847C; p.99R; p.120D; p.126L; g.21081C; p.371D; p.377V; p.439H; g.24762C]	0.6±0.4	1.8±0.9	1.9±0.8
*2A1	[g.4847C; p.99R; p.120D; p.126P; g.21081C; p.371D; p.377V; p.439R; g.24762T]	0.4±0.3	1.4±0.8	2.6±0.9
*2A2-l	[g.4847C; p.99R; p.120D; p.126P; g.21081C; p.371D; p.377A; p.439R; g.24762T]	4.6±1.0	2.7±1.0	2.2±0.8
*2B1-i	[g.4847C; p.99R; p.120D; p.126P; g.21081C; p.371Y; p.377V; p.439R; g.24762T]	5.6±1.0	10.8±2.1	11.5±1.8
*2B2A-i	[g.4847A; p.99R; p.120D; p.126P; g.21081C; p.371Y; p.377V; p.439R; g.24762T]	65.4±2.2	59.9±3.3	59±2.8
*2B2B-l	[g.4847A; p.99R; p.120G; p.126P; g.21081C; p.371Y; p.377V; p.439R; g.24762T]	1.5±0.6	2.7±1.1	1.3±0.6
*2B2A-i. 1B1-h	**[g.4847A; p.99R; p.120D; p.126P; g.21081T; p.371D; p.377V; p.439R; g.24762C]**	**0.2±0.2**	**0**	**0**

a : phylogenetic nomenclature according to [26] and [38], where “h”, “ï” and “l” refer to *MASP2* haplotypes associated with low (<200 ng/ml), intermediary (200–600 ng/ml) and high (≥600 ng/ml) MASP-2 levels (cut off adopted by [26] and [35]),

b : reference sequence NT_021937 (GenBank sequence); anti-CCP: anti-cyclic citrullinated peptide antibodies; n: number of chromosomes; SD: standard deviation. In bold: recombinant haplotype.

**Figure 2:**
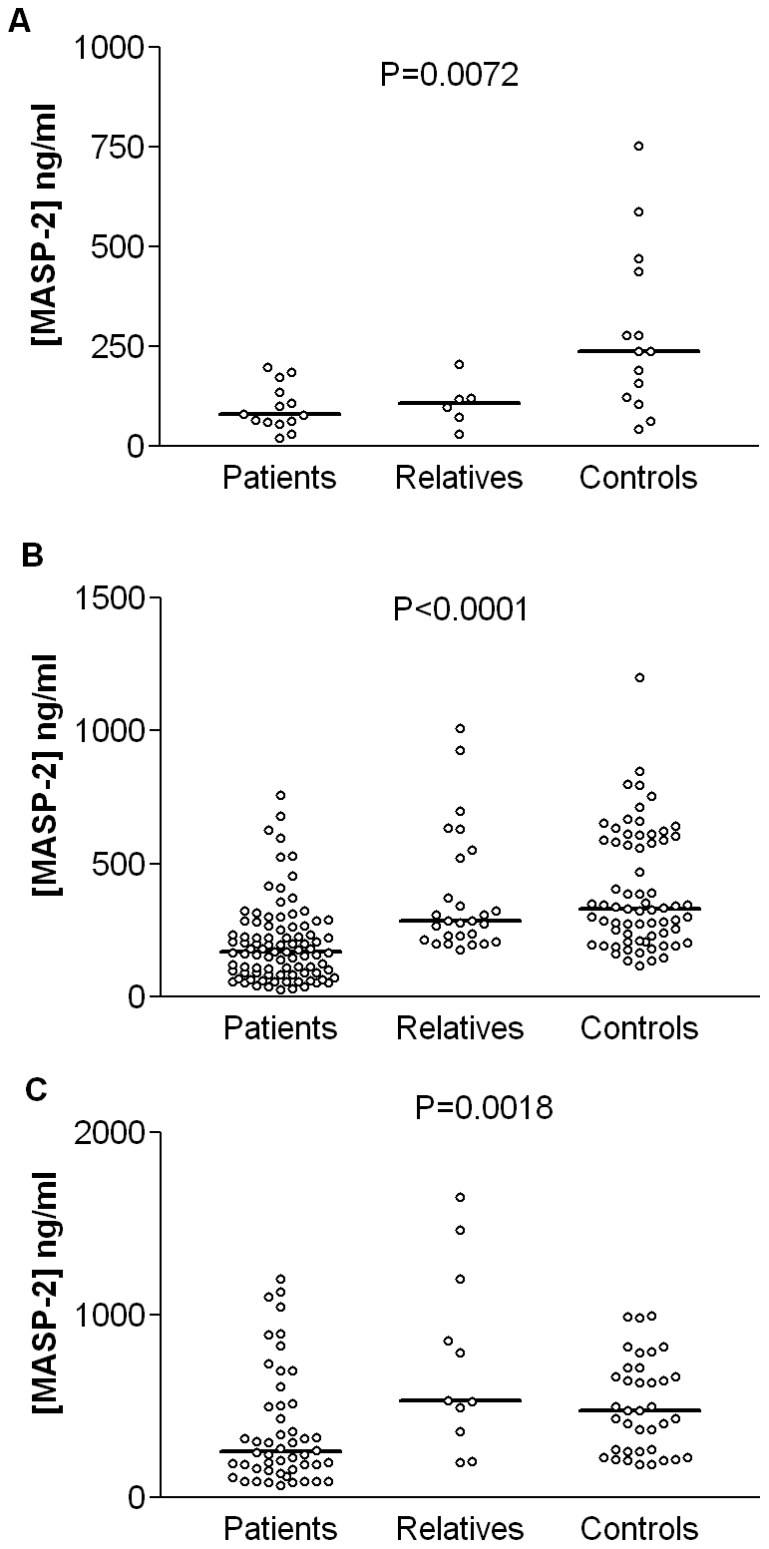
MASP-2 levels in RA patients, relatives and controls according to haplotype producing profiles. Data is shown with medians, differences were calculated with Kruskal-Wallis test A: Low MASP-2 producing haplotypes (*2B2B-l/*1A, *2B2A-i/*2B2B-l, *2B2B-l/2A1*, *2B2A-i/*2A2-l, *2B1-i/2A2-l*, *2B1-i/*1C2-l, *1C2-l/*1C2-l, *2B2A-i/*1C2-l, *2B2B-l/*1C2-l, *2A2-l/*1C2-l); B: Intermediary MASP-2 producing haplotypes (*2B2A-i/*2B2A-i, *2B2A-i/*1A, *2B2A-i/*2B1-i, *2B1-i/*1A, *2B1-i/2A1, *2B1-i/*2B1-i); C: High MASP-2 producing haplotypes (*2B2A-i/*1B1-h, *2B2A-i/*1B2-h, *2B1-i/*1B1-h, *1B1-h/*1B1-h, *1A/*1B1-h, *2A1/1B1-h, *2A1/*2A1, *2B2A-i/*2A1, *2A1/*1A, *2B2A-i.1B1-h/1B1-h).

There was, as expected, a significant association between MASP-2 levels and high, intermediary and low MASP-2-producing genotypes in all investigated groups, but patient MASP-2 levels were always lower compared to all other groups (Figure 2). Two haplotypes were associated with three times increased susceptibility to RA: *2A1 and *2B1-i, of which the last one is associated with intermediary MASP-2 levels (44/312 or 14.1% in the patients vs. 28/460 or 6.1% in the controls, binary logistic regression P = 0.004, Tables 2 and 3). There was no association between *MASP2* haplotypes and clinical characteristics such as time of disease duration, functional class and extra-articular manifestations (data not shown).

The deficiency-causing *p.120G* and/or *p.439H* amino acid substitutions (embedded in the *2B2B-l and *1C2-l haplotypes, respectively), were associated with five times increased susceptibility to articular symptoms among relatives, independently of age, gender, ethnic group, smoking habit and anti-CCP and rheumatoid factor positivity (in relatives with and without articular symptoms, 5/50 or 10% vs. 5/172 or 2.9%, respectively, binary logistic regression P = 0.02, Table 3).

## Discussion

RA is a complex and still not entirely understood disease. Environmental, genetic and immunological factors are known to contribute for its development, however the identification of components that determine disease establishment remains a great challenge [39], [40]. Although the complement system has already been related to RA development and clinical presentation [41], [42], this is, to our knowledge, the first study to examine the association between the central serine protease of the lectin pathway of complement, MASP-2, and human RA.

In this study, high MASP-2 levels decreased the susceptibility to RA and to the onset of articular symptoms among patient relatives. Protection against RA was independent of classical markers of disease establishment and progression such as rheumatoid factor and anti-CCP antibodies. More surprising, these well-established markers did not show any significant association with articular symptoms. Since relatives from RA patients are characterized as a risk group for the development of RA [9], this leads us to suggest that MASP-2 levels might be used as an independent biomarker to follow-up individuals with familiar RA predisposition.

In the present study, MASP-2 levels in patients were almost always lower than in controls and relatives, independently from genotypes. A possible explanation for lower MASP-2 levels in RA patients could be related to protein consumption and/or lower gene expression, also described by Ip et al (2000), which found similar results for MBL in the southern Chinese [41]. Reduced MASP-2 levels due to protein consumption were also recently associated with ischemia-related necrotic myocardial injury in a human cohort [31]. On the other hand, *MASP2* expression may be impaired in detriment to the expression of other proteins such as MBL-associated protein of 19 kDa, an alternative splice product of the *MASP2* gene [26]. Concomitantly, the synovial permeability of joints is slightly augmented in RA, which enhances protein diffusion from plasma into synovial fluid. In addition, the synovial fluid/plasma ratio of complement proteins have been already described to be higher in RA in comparison to other arthropathies, suggesting a sequestration and consumption of complement proteins in the synovial fluid in RA patients [18].

Studies have shown that cytokines such as interleukin 6 and the binding protein STAT3 can modify the expression of *MASP2* genes [43], [44]. Interleukin 6 is highly expressed by synoviocytes in RA patients and has been shown to down regulate the expression of complement system proteins, including MASP-2 [43], [45]. Thus, the consequences of the interaction between pro-inflammatory cytokines, such as interleukin 6, and MASP-2 might play an important role on the development of RA.

The reduction of MASP-2 serum levels in RA patients could also be due to hormonal alterations, such as from thyroid and growth hormones, and acute phase response, since the expression of some complement proteins can be influenced by these conditions [46], [47].

In the present study, the *2A1 and *2B1-i MASP2 haplotypes increased susceptibility to RA, which might be rather the result of a variant linked to these haplotypes. On the other hand, the deficiency-associated variants *p.120G* and *p.439H* (*2B2B-l and *1C2-l, respectively), which turn mature MASP-2 proteins unable to activate complement, increase the susceptibility to articular symptoms in individuals with familiar RA predisposition. This leads us to suggest that the ability to activate the lectin pathway of complement might play a critical role on disease onset, which is in accordance with other studies pointing to the importance of complement deficiency in the immunopathology of RA [41], [42]. In Norwegian patients with juvenile rheumatoid arthritis, MBL deficiency was found associated with early disease onset and was also shown to favor disease remission, pointing a dual role of this protein in this rheumatic disease [48]. Further studies should clarify if MASP-2 follows this trend as well.

## Conclusion

In this study, we found the first evidence that MASP-2 deficiency might play an important role in the development of RA and articular symptoms among relatives of RA patients.
